# Effects of nitroglycerine on coronary flow velocity before and during adenosine provocation

**DOI:** 10.1186/s12947-016-0091-2

**Published:** 2016-12-06

**Authors:** Ann Wittfeldt, Anders Jeppsson, Li-Ming Gan

**Affiliations:** 1 Dept of Molecular and Clinical Medicine, Institute of Medicine at Sahlgrenska Academy, University of Gothenburg, 41345 Gothenburg, Sweden; 2 Department of Cardiology, Sahlgrenska University Hospital, 41345 Gothenburg, Sweden; 3 Department of Cardiothoracic Surgery, Sahlgrenska University Hospital, 41345 Gothenburg, Sweden; 4 AstraZeneca R & D, 43150 Molndal, Sweden

**Keywords:** Ultrasound, Non-invasive, Coronary flow velocity reserve, Nitroglycerine

## Abstract

**Background:**

Transthoracic echocardiography-assessed coronary flow velocity reserve (CFVR) evaluates coronary microvascular arterial function. Coronary flow velocity measurements at baseline and during hyperemia are used to calculate CFVR. Adenosine infusion induces hyperemia but it is not known if it causes a maximal response. We hypothesized that pre-treatment with nitroglycerine before adenosine provocation enhances hyperemia.

**Methods:**

Twenty-three healthy study subjects (mean age 27.5 ± 5.5, 35% women) underwent CFVR measurements before and after pretreatment with sublingual nitroglycerine (0.5 mg). Hyperemia was induced by adenosine infusion (140 μg/kg/min). In addition, the effect of nitroglycerin on left main coronary artery diameter was assessed.

**Results:**

Pretreatment with nitroglycerine increased median CFVR from 3.6 (range 2.8–4.3) to 5.0 (4.1–6.0), *p* = 0.002. The increase was caused by a marked reduction in baseline coronary flow velocity 17 (15–24) vs 27 (19–31) cm/s, *p* < 0.0001) while hyperemic velocity remained unchanged (90 (68–116) vs 93 (75–105) cm/s, *p* = 0.48). Nitroglycerin significantly dilated the left main coronary artery (from median 3.1 (2.7–3.6) mm to 3.8 (3.1–4.3) mm, *p* = 0.018).

**Conclusion:**

Pretreatment with nitroglycerine dilates coronary arteries and increases coronary flow velocity reserve indicating that adenosine alone causes a submaximal hyperemia.

## Background

Transthoracic color Doppler echocardiography-assessed coronary flow velocity reserve (CFVR) is an emerging non-invasive method to quantify coronary microvascular function and evaluate volumetric coronary flow reserve (CFR) [1–4]. CFVR has opened up new possibilities to explore cardiovascular physiology and to evaluate responses to treatment, due to its good reproducibility and low method-dependent variability if performed by well-trained operators [5–7], and it has been shown that low CFVR predicts epicardial coronary artery stenosis [8–10] and cardiovascular events in different patient populations [11–13]. CFVR is suitable for repeated measurements of coronary function since it is non-invasive, without radiation, easily accessed with a clinical ultrasound device and is associated with acceptable discomfort to the patient. With modern ultrasound platforms it is also possible to non- invasively evaluate size and morphology of small structures like certain segments of coronary arteries [14, 15].

Mean coronary flow velocity at baseline and during maximum hyperemia are used to calculate CFVR. Infusion of the vasodilator adenosine is an established method to induce hyperemia [16] and studies has suggested 140 μg/kg/min as suitable dose [17]. Adenosine acts mainly on small resistance vessels, but influences also to a lesser degree epicardial arteries [18]. Even small changes in epicardial vessel diameter would result in alterations of flow velocity [19]. Thus, it is yet not known whether CFVR due to adenosine-induced dilatation of epicardial vessels underestimate CFR, which is a volumetric flow reserve.

It is possible that other vasodilators alone or in combination with adenosine would induce an even higher level of hyperemia [19]. Nitroglycerin (NTG) is a vasodilator mainly acting on epicardial arteries [20, 21]. NTG may be used in combination with adenosine during measurements of flow fraction reserve in connection to coronary angiogram in patients with coronary artery disease. In this context NTG is in combination with adenosine used to maximize blood flow and prevent wire induced artery spasm.

We hypothesized that pre-treatment with nitroglycerine before adenosine provocation in CFVR measurements in healthy volunteers enhances the hyperemia further in comparison to adenosine alone, due to maximum dilatation of not only the resistance vessels but also epicardial arteries. In addition, we explored the effects of nitroglycerine on basal coronary flow and coronary artery diameter and assessed the reproducibility of repeated CFVR measurements using adenosine infusion with a short wash out period.

## Methods

### Study subjects

Twenty-three healthy volunteers (mean age 27.5 ± 5.5 years, 38% women) participated in the study. Inclusion criteria were age 18–40 years, normal BMI and no chronical or acute illness requiring medication. Exclusion criteria were history of asthma, chronic obstructive pulmonary disease, anxiety disorder, and ongoing infection. Study subjects characteristics are presented in Table 1.

**Table 1:** Study subjects characteristics. Number and percentage or mean and standard deviation

Gender (female)	8 (35%)
Age (years)	27.5 ± 5.5
Weight (kg)	70.5 ± 9.4
Length (cm)	177.5 ± 7.9
Body mass index (kg/m^2^)	22.3 ± 2.2
White blood cell count (×10^9^/L)	5.7 ± 1.1
Hemoglobin concentration (g/L)	141 ± 13
Left ventricular ejection fraction (%)	
At rest	61 ± 6
During adenosine	66 ± 5
Heart rate (beats per minute	
At rest	59 ± 10
During adenosine	84 ± 13

### Study design

All study subjects were investigated on two consecutive days. The study protocol on each study day included two sets of coronary flow velocity measurements at baseline and during adenosine infusion. On the study days all subjects were fasting for four hours and were caffeine free for at least 24 h before the investigation. On day 1 a suitable vessel segment of mid left anterior descending artery (LAD) regarding measurement quality and breathing interference was identified with transthoracic color doppler echocardiography. During the first measurement (1:1), baseline coronary flow velocity was recorded and thereafter adenosine infusion at a rate of 140 μg/kg/min was started. Hyperemic coronary flow measurements were obtained during 5 min where the highest recordings were registered. After completion of the measurements, the adenosine infusion was paused. After a ten minute washout period a new measurement period was started (1:2) when new baseline measurements were obtained before the adenosine infusion was re-started at the same rate for new hyperemic measurements. Heart rate and systolic blood pressure were recorded every minute during adenosine infusion to detect potential adverse reactions.

On day 2, two new measurements were performed (2:1 and 2:2). The measurements were identical to the procedures on day 1 with the exception that after five minutes of the washout period between the two sets of measurements 0.5 mg of sublingual nitroglycerine (Recip, Jordbro, Sweden) was administrated to the study subjects. Five minutes after nitroglycerine administration new baseline measurements and adenosine–induced hyperemic measurements were performed (period 2:2). In a subgroup of nine subjects with good visualization of the left main coronary artery, the diameter of the vessel was measured before and after nitroglycerine administration.

### Coronary blood flow velocity measurements

**Fig. 1:**
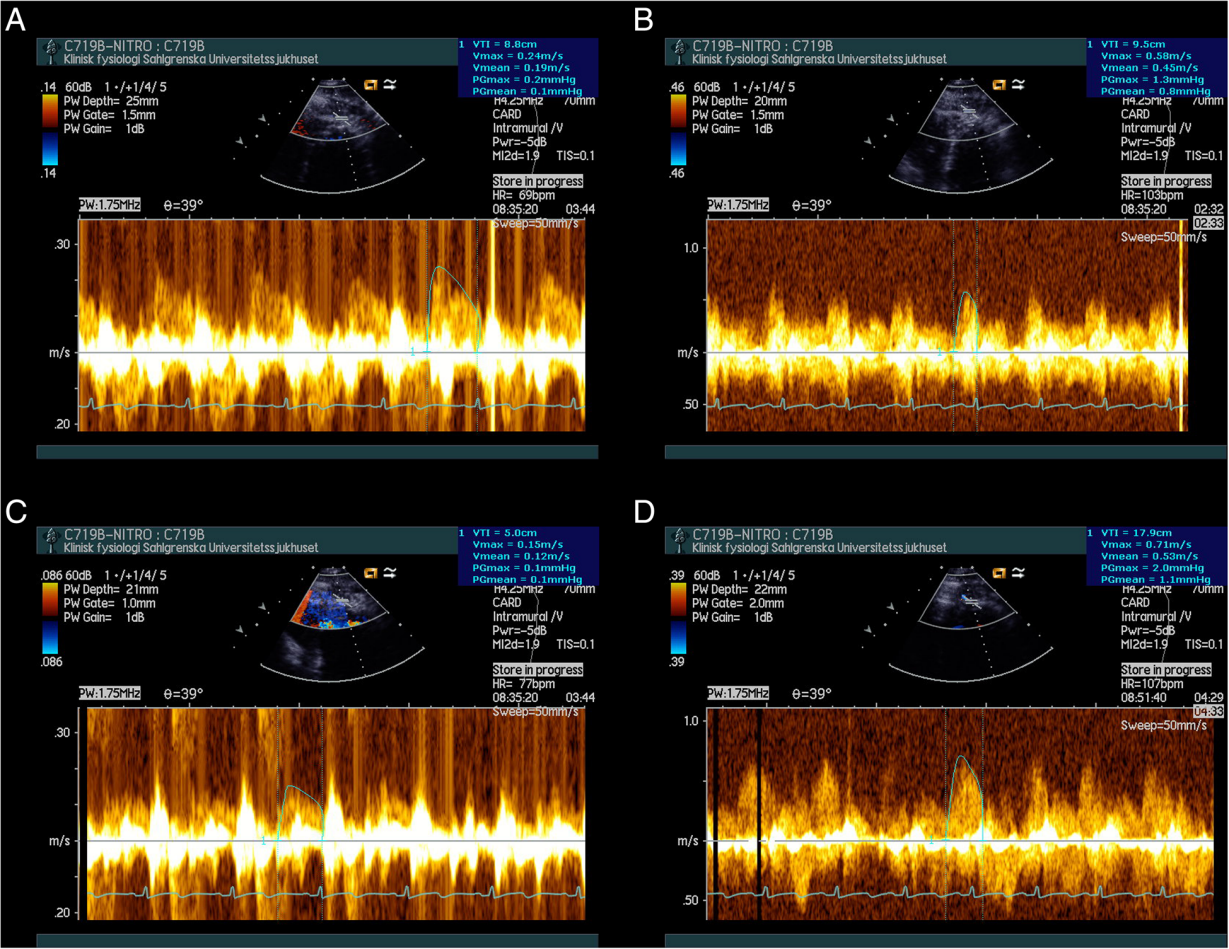
Recording of coronary flow velocities in an individual study subject. The velocities were recorded at baseline (**a**), at adenosine-induced hyperemia (**b**), at baseline with nitroglycerine (**c**), and at adenosine-induced hyperemia with nitroglycerine (**d**)

A Siemens Acuson platform equipped with a 4V1C transducer with 3.5 MHz color Doppler frequency and 1.75 MHz spectral Doppler frequency (Siemens, Acuson Sequoia 512, Mountainview) was used to measure coronary flow velocity (CFV) in the mid to distal left anterior descending coronary artery (LAD), in a slightly modified apical two-chamber view. The same operator performed all measurements to ensure minimum variability. CINE-loops and Doppler images were stored for offline analysis using Tomtec image analysis software (Image Arena 2.9.1, Tomtec Imaging Systems, GmbH, Unterschleissheim, Germany). CFVR was calculated as the ratio between mean hyperemic CFV and mean baseline CFV. A typical recording of coronary flow velocity at baseline without nitroglycerine is shown in Fig. 1.

### Diameter of the left main coronary artery

The left main coronary artery was visualized in a modified short axis view at the level of aortic root and a CINE-loop of a full cardiac circle with a frame rate up to 70 frames/s was stored for offline measurements. The diameter was measured as close as possible to the R- wave and at the same location both at baseline and after nitroglycerine administration.

### Statistics

No data for a sample size calculation was available before study start. The number of included subjects are based on own experiences and similar studies. Data was not normally distributed. Data are thus presented as median and range. Wilcoxon matched paired test was used to compare mean CFV, CFVR and artery diameter before and after adenosine and nitroglycerine administration. Friedman’s test was used to compare the two baseline measurements on day 1 and the baseline measurement on day 2.

## Results

### General

Twenty of the 23 subjects completed all four measurements. One was excluded due to anxiety during adenosine infusion and two were excluded due to difficulties to get satisfying CFV measurements after NTG administration, due to low CFV.

### Baseline flow velocity

There was no significant difference in flow velocity between the two baseline measurements on day one (1:1 and 1:2) and the first baseline measurement on day two (2:1), *p* = 0.82, Table 2. In contrast, baseline flow velocity was significantly lower after nitroglycerine administration (2:2) in comparison to the measurement before nitroglycerine (2:1) and to the two measurements on day 1, *p* < 0.001, Table 2.

**Table 2:** Coronary flow velocity at baseline and during adenosine-induced hyperemia. Median and range

Measurement period	Baseline velocity (cm/s)	Velocity during hyperemia (cm/s)
Day 1, measurement 1 (1:1)	25 (20–31)	87 (72–93)
Day 1, measurement 2 (1:2)	25 (19–31)	97 (77–111)^#^
Day 2, measurement 1 (2:1)	27 (19–31)	93 (75–105)
Day 2, measurement 2 (after nitroglycerine) (2:2)	17 (15–24)***	90 (68–116)

*** = *p* < 0.001 vs measurement 1:1, 1:2 and 2:1; # = *p* < 0.05 vs measurement 1:1

### Coronary flow velocity during hyperemia

Maximum velocity during hyperemia was higher during the second measurement period on day 1 (1:2) compared to the first measurement the same day (1:1), *p* = 0.021), Table 2. No significant differences in maximum velocity during hyperemia were observed between the first measurement on day 1 (1:1) and the first measurement day 2 (2:1), *p* = 0.20. Maximum velocity during nitroglycerine administration (measurement 2:2) did not differ significantly compared with the measurement without nitroglycerine on the same day (2:1) or to the hyperemic measurements on the first day (1:1 and 1:2), *p* = 0.53, Table 2.

### Coronary flow velocity reserve

**Fig. 2:**
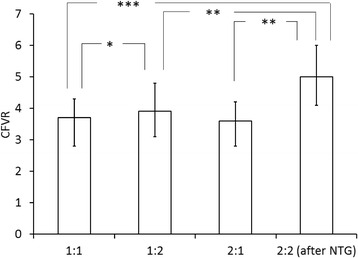
Coronary flow velocity reserve (CFVR) at baseline and during adenosine-induced hyperemia. NTG = nitroglycerine. * = *p* < 0.05, ** = *p* < 0.01, *** = *p* < 0.001

CFVR during the different measurement periods are shown in Fig. 2. There was no significant difference in CFVR when comparing the first measurements on each study day (measurement 1:1 and 2:1), 3.7 (2.3–7.2) vs. 3.6 (2.3–6.9), (*p* = 0.39). CFVR after nitroglycerine administration (measurement 2:2) was significantly higher than with only adenosine (measurement 2:1), 5.0 (2.4–9.2) vs 3.6 (2.3–6.9), (*p* = 0.002) and significantly higher than at the two measurements on day 1, 3.7 (2.3–7.2), (*p* < 0.001) and 3.9 (2.5–7.3), (*p* = 0.007), respectively. CFVR was significantly higher during the second measurement on day 1 (1:2) than during the first measurement the same day (1:1), (*p* = 0.018).

### Effects of nitroglycerine on vessel diameter

**Fig. 3:**
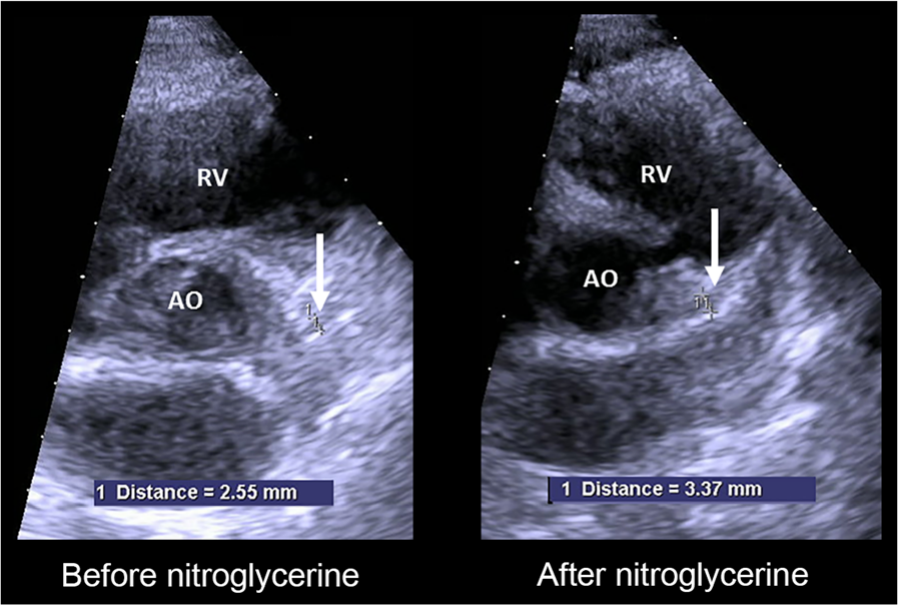
Left main coronary artery diameter before and after nitroglycerine administration

Left main coronary artery dilated significantly after nitroglycerine administration (from median 3.1 (2.7–3.6) mm to 3.8 (3.1–4.3) mm, *p* = 0.018), Fig. 3.

## Discussion

The main findings of the present study were that pre-treatment with nitroglycerine before adenosine-induced hyperemia significantly increased coronary flow velocity reserve.

In the present study we hypothesized that pre-treatment with nitroglycerine before adenosine provocation in CFVR measurements would enhance the hyperemia further in comparison to adenosine alone. This hypothesis was based on the known vasodilating effects of nitroglycerine on epicardial arteries and clinical experiences from fractional flow reserve (FFR) measurements and myocardial scintigraphy where pretreatment with NTG may be used to enhance hyperemia [20–22].

After administration of NTG, there was a reduction in baseline coronary flow velocity (before hyperemia) by approximately 30%. This reduction in flow velocity is more likely a result of a dilatation of the epicardial arteries, than due to a reduction of total blood flow, since the same amount of blood at a constant flow in a larger vessel leads to a reduction in velocity. The effect on vessel diameter is supported by the present study where the diameter of the left main coronary artery increased with approximately 20% after NG administration. Our baseline results thus confirm the results shown by Takagi et al. [21] where they, using the same methodology, found that NTG administration caused a decrease in coronary resting flow velocity and a dilatation of epicardial arteries.

During adenosine provocation after NTG administration one would expect a reduction of coronary blood flow velocity, compared to adenosine provocation without NTG, since NTG dilates epicardial vessels. Contrary to this hypothesis, there was no significant change in hyperemic coronary blood flow velocity when compared to the measurement without nitroglycerine. This may be interpreted as an increase in total blood flow through the myocardium after the combination of NTG and adenosine administration, compared to only adenosine. Thus, our results suggest that NTG pretreatment increased adenosine-induced coronary blood flow in healthy volunteers, supporting that epicardial vessel dilation is essential to cause maximal hyperemic flow.

The reduced baseline velocity resulted in a significantly increased CFVR even though nitroglycerine did not increase hyperemic blood flow velocity (Table 2). From a methodological point of view, CFVR is a good estimate of CFR only when the epicardial vessel diameter is maintained before and during adenosine infusion. In case epicardial vessels dilate in response to increased shear stress e.g., following inceased flow during adenosine challenge, CFV increase measured at the same epicardial vessel site as at baseline may be underestimating the true flow increase. This indicates that CFVR with nitroglycerine in combination with adenosine might give a better volumetric coronary flow reserve (CFR) estimation and a more stable measurement routine than hyperemia induced by adenosine alone [23].

The results of the present study also demonstrate that the reproducibility of flow velocity measurements is satisfactory since the baseline measurements both at the same day and between the two study days did not differ significantly. This indicates that non.invasive flow velocity measurements are stable and can be used to detect even relatively small changes in flow velocity over time. A low intraindividual variability over time is a key factor for using this technique to evaluate possible changes in coronary flow. However, there was one potentially important exception. The flow velocity during adenosine-induced hyperemia was significantly higher during the second measurement period on day 1 in comparison to the first measurement the same day. The wash-out period between the two measurements was only 10 min and it is possible that the effects had not fully ceased, even though the half life of adenosine is only a few seconds. Alternative explanations include upregulation of adenosine receptors or increased sympathicus drive. The latter explanation may be less likely since basal flow and heart rate was unaltered between the two measurements. Independently of the reason, a longer washout period than 10 min can be recommended if repeated adenosine- induced hyperemic periods are required.

### Limitations

The present study had some limitations. The size of the study population was limited. In particular, left main diameter was measured only in eight patients. This may reduce the validity of our observation. However, the 20% difference between left main diameter before and after nitroglycerine administration is within what previously have been reported (8–25%) [21, 24]. A further limitation is that the off line measurements were not blinded to the operator. Finally, the study was performed in a young, healthy population with healthy coronary arteries and it is thus unclear whether the results can be applied on patients with cardiac disease. A prospective study with a larger number of subjects in a relevant patient population with manifested coronary artery disease would therefore be desirable.

## Conclusion

Pre-treatment with nitroglycerine before adenosine infusion could be a possible way to minimize the difference between CFVR and CFR. It could also ensure maximal coronary artery dilatation resulting in a more accurate way of evaluating maximal coronary flow reserve.

## Abbrevations

CFR: Coronary flow reserve
CFV: Coronary flow velocity
CFVR: Coronary flow velocity reserve
LAD: Left anterior descending artery
NTG: Nitroglycerine
